# Microstructure and Mechanical Performance of PBF-LB/M 316L Stainless Steel

**DOI:** 10.3390/ma18122720

**Published:** 2025-06-10

**Authors:** Haoyu Cai, Renche Wang, Tao Wang, Shuaishuai Du, Molin Su

**Affiliations:** 1 Nanjing Research Institute of Electronics Technology, Nanjing 210039, China; washuis@163.com (H.C.);; 2 CNPC Research Institute of Safety & Environment Technology, Beijing 102206, China

**Keywords:** tensile fracture, plastic deformation, additive manufacturing, microstructures, stainless steel

## Abstract

The laser-based powder bed fusion of metal (PBF-LB/M) process of 316L stainless steel (SS) was systematically investigated under varying scanning spacings to assess its microstructural and mechanical properties. Optimized laser parameters were employed, and the resulting microstructure and mechanical performance were thoroughly characterized through surface and cross-sectional scanning electron microscopy (SEM), electron backscatter diffraction (EBSD) analysis, fracture surface examination, and tensile testing. The results indicated that a scanning spacing of 0.11 mm produced the most favorable mechanical properties, characterized by a dense microstructure and refined grain morphology. These findings provide critical insights for the optimization of PBF-LB/M process parameters, contributing to the advancement of additive manufacturing techniques for 316L SS.

## 1. Introduction

Laser-based powder bed fusion of metal (PBF-LB/M), a subset of powder bed fusion technologies, was found to have revolutionized additive manufacturing (AM) by enabling the direct production of complex geometries from metal powders [1,2,3]. This layer-by-layer process was carried out using a high-power laser to selectively fuse powder particles according to a digital design, allowing for the creation of intricate internal structures and near-net-shape components that were challenging to achieve using traditional manufacturing methods. By spreading the energy of the laser beam over two spots [4], additional heat was introduced into the weld of the work of Kovačócy, which ensured a slowdown in the cooling rate of the SS and possibly ensures the desired ratio of ferrite and austenite in the microstructure. Among various materials, 316L stainless steel (SS) was widely used in PBF-LB/M due to its excellent corrosion resistance, mechanical properties, and biocompatibility, making it suitable for applications in the biomedical, aerospace, and automotive sectors [5,6,7,8,9]. In biomedicine, the results could benefit the development of customized 316L SS bone implants via PBF-LB/M, where its biocompatibility and the technology’s ability to fabricate porous structures for osseointegration are critical. In the automotive industry, the findings may support the production of high-strength, corrosion-resistant 316L SS lightweight chassis parts through PBF-LB/M, enhancing fuel efficiency and performance. However, the final properties of PBF-LB/M 316L SS were highly sensitive to process parameters [10,11,12], which directly influenced the microstructure, density, and mechanical performance of the material [13].

Key parameters in PBF-LB/M, such as laser power [14,15,16], scan speed [17,18,19], layer thickness [20], and scan spacing, were found to play a crucial role in controlling the thermal history during the melting and solidification processes [21]. These factors influenced the stability of the melt pool, the degree of fusion between adjacent layers, and, ultimately, the formation of defects such as porosity, residual stresses, and anisotropic grain structures. While laser power and scan speed have been extensively studied, scan spacing—the distance between consecutive laser scan lines—received comparatively less attention [22,23,24]. Scan spacing directly impacted the energy input per unit area and, consequently, the overlap between adjacent melt pools, which significantly affected the part’s density and mechanical properties [25].

Cai et al. [26] tested large hatch spacings (2~4 mm) in robotic laser AM, finding that 2.5 mm spacing yielded superior mechanical properties: a tensile strength of 597 MPa, an elongation of 45.8%, and >99% density. This was attributed to reduced thermal stress and uniform layer deposition. However, spacings >3 mm disrupted interlayer fusion, lowering strength by 18%. Stephenson et al. [27] reported that Dehoff point-filling strategies mitigated residual stress in TC4 titanium, but hatch spacing variations (0.07~0.15 mm) still dominated porosity trends. ANSYS 16.0 Version simulations corroborated this, showing that spacings >0.15 mm exceeded the 0.5% porosity threshold due to insufficient overlap.

An optimal scan spacing was found to ensure sufficient overlap between melt tracks, promoting full densification and minimizing void formation. However, excessive overlap (resulting from small scan spacing) could lead to overheating, residual stresses, and finer grain structures, while insufficient overlap (due to large scan spacing) could result in unmolten regions, increased porosity, and reduced mechanical strength. These effects were particularly pertinent for applications requiring high structural integrity, as voids and defects acted as stress concentrators, compromising fatigue resistance and tensile strength [28]. Therefore, understanding the influence of scan spacing on both the microstructure and mechanical properties of 316L SS was deemed essential for optimizing PBF-LB/M parameters and achieving high-performance parts.

Based on this study [29], the effects of three different scan spacings (0.07 mm, 0.11 mm, and 0.15 mm) on the microstructural and mechanical properties of PBF-LB/M 316L SS were investigated. SEM imaging was employed to analyze both surface and cross-sectional microstructures, while EBSD was used to provide insights into grain orientation and texture. Tensile testing was performed to evaluate the mechanical performance, and fracture analysis was conducted to offer a deeper understanding of the fracture mechanisms associated with each scan spacing. The findings from this study aimed to elucidate the role of scan spacing in enhancing the structural integrity and mechanical performance of PBF-LB/M 316L SS, providing valuable insights for industrial applications where optimized mechanical properties were critical.

## 2. Experimental Section

316L SS samples were fabricated using the PBF-LB/M process with a laser power of 200 W, an exposure time of 80 µs, and scan spacings of 0.07 mm, 0.11 mm, and 0.15 mm. The layer thickness was maintained at 50 µm, and a preheat temperature of 100 °C was applied. A scan rotation angle of 67° between layers was implemented to minimize anisotropy.

SEM (Sigma 30, Carl Zeiss, Oberkochen, Germany) was used to analyze surface and cross-sectional microstructures, while the QUANTAX EBSD device (Bruker, Ltd., Billerica, MA, USA) provided grain orientation and texture information. Tensile testing was conducted to evaluate mechanical properties, with specific attention to the influence of scan spacing. Fractographic analysis was performed on fractured tensile specimens to understand failure modes associated with different scan spacings.

**Figure 1:**
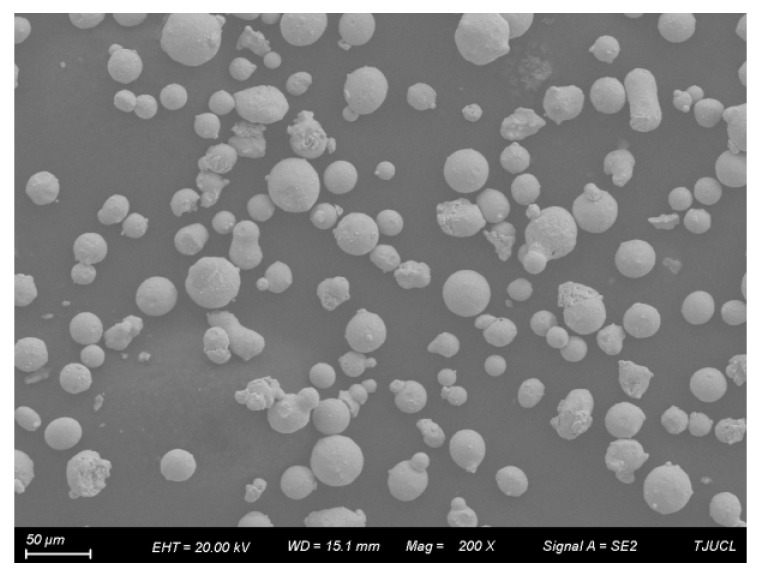
SEM images of powder for PBF-LB/M 316 SS.

The 316L SS powder used in this study was characterized by SEM to evaluate particle morphology and size distribution. The SEM images revealed that the powder particles exhibited a nearly spherical morphology, which was critical for ensuring uniform powder spreading during the PBF-LB/M process. A minimal presence of satellite particles was observed, indicating good flowability and packing density. The particle size distribution ranged between 10 and 20 µm, as shown in Figure 1. These characteristics contributed to consistent layer thickness and enhanced laser absorption, promoting optimal melting and densification during fabrication.

Tensile experiments were conducted on PBF-LB/M specimens manufactured with scan spacings of 0.07, 0.11, and 0.15, with a repetition rate of three times for each process. The tensile tests were performed on the DDL300 tensile tester (Sinotest Equipment Co., Ltd., Changchun, China) using a crosshead speed of 1.5 mm/min, following the guidelines of GB/T-228 [30]. The grain size in this paper was the average equivalent grain diameter size, and the grain size distribution was the area-weighted grain size distribution, which excluded the boundary grains. The average grain size was obtained via the Esprit 2.1 software of the QUANTAX EBSD device (Bruker, Ltd.), and the statistical process referred to the standard ASTM E2627 [31].

## 3. Result and Discussion

### 3.1. SEM Analysis

#### 3.1.1. Cross-Sectional SEM Analysis

**Figure 2:**
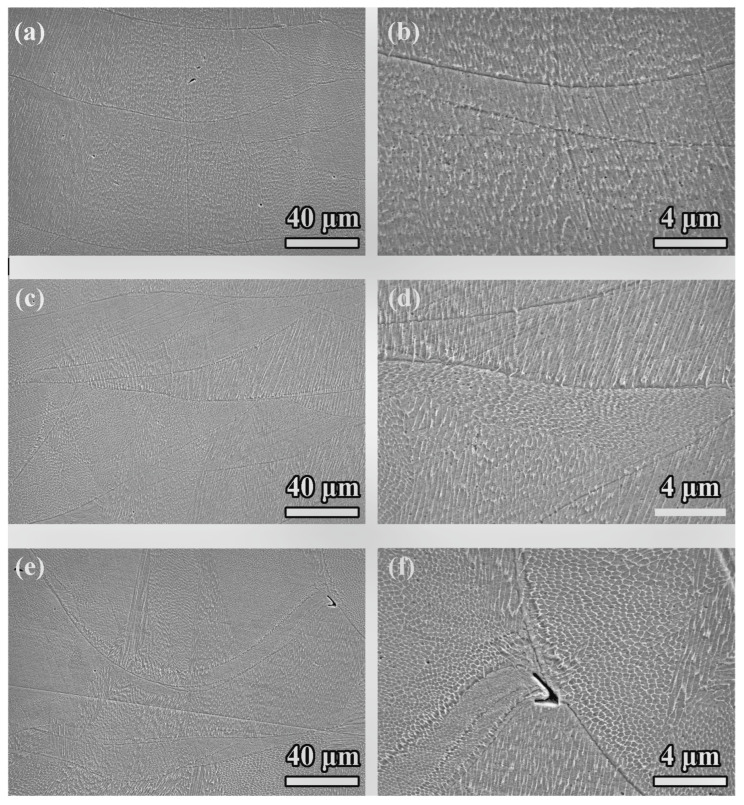
Cross-sectional SEM images of PBF-LB/M 316 SS with scan spacings of 0.07 mm: (**a**) section and (**b**) overlap zone; 0.11 mm: (**c**) section and (**d**) overlap zone; 0.15 mm: (**e**) section and (**f**) overlap zone.

The cross-sectional SEM images of samples fabricated with scan spacings of 0.07 mm (see Figure 2a,b), 0.11 mm (see Figure 2c,d), and 0.15 mm (see Figure 2e,f) revealed a generally consistent microstructure across all conditions, with no substantial differences observed in terms of grain morphology or defect distribution. The grains exhibited a predominantly columnar structure oriented along the build direction, which was typical of PBF-LB/M due to the directional solidification caused by the steep thermal gradients during the process.

Although minimal porosity was detected in all samples, no significant trends or distinguishing features were evident in the cross-sections that could clearly correlate with the scan spacing variations. This lack of apparent differences suggested that the cross-sectional analysis alone might not have adequately captured the subtle effects of scan spacing on microstructural evolution.

#### 3.1.2. Surface-Sectional SEM Analysis

**Figure 3:**
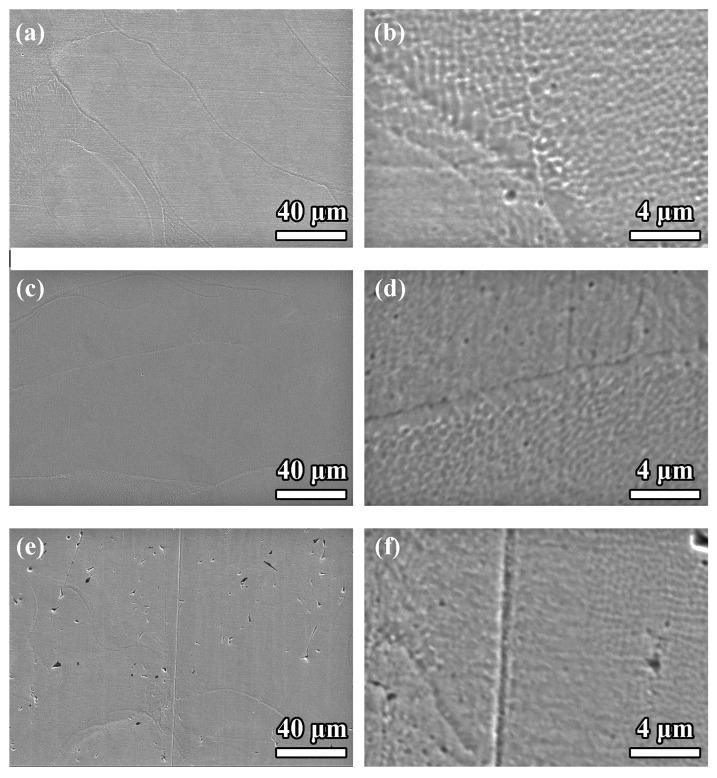
Surface-sectional SEM images of PBF-LB/M 316 SS with scan spacings of 0.07 mm: (**a**) surface and (**b**) overlap zone; 0.11 mm: (**c**) surface and (**d**) overlap zone; 0.15 mm: (**e**) surface and (**f**) overlap zone.

As shown in Figure 3a,b, the SEM images of the surface produced at a 0.07 mm scan spacing reveal a relatively uniform morphology characterized by closely spaced laser tracks and minimal porosity. However, localized inconsistencies were observed, particularly along the melt pool boundaries, which were slightly irregular due to excessive energy density resulting from significant track overlap. While the high degree of overlap facilitated effective fusion, it also introduced surface roughness and potential thermal stress concentration zones.

The surface morphology observed for the 0.11 mm scan spacing is shown in Figure 3c,d, indicating an optimal balance between energy density and track overlap. The SEM images reveal a highly uniform and smooth surface with negligible defects such as pores or surface irregularities. The melt pool boundaries were well defined and continuous, reflecting stable and consistent laser–material interaction. The reduced surface roughness and absence of significant defects suggested that the 0.11 mm scan spacing effectively minimized thermal stresses while maintaining sufficient energy input for complete melting and fusion.

As shown in Figure 3e,f, the surface morphology for the 0.15 mm scan spacing exhibited clear signs of insufficient fusion, as evidenced by the presence of pores, unmelted powder particles, and irregular laser track continuity. The reduced overlap between adjacent tracks resulted in incomplete melting, leading to surface defects that could have acted as stress concentrators under mechanical loading.

The comparison of SEM surface morphologies clearly indicated that the 0.11 mm scan spacing provided the best balance of laser track overlap and energy input, resulting in a smooth, uniform, and defect-free surface. In contrast, the 0.07 mm spacing suffered from minor irregularities due to excessive energy density, while the 0.15 mm spacing exhibited significant defects caused by insufficient track overlap.

### 3.2. EBSD Analysis

#### 3.2.1. Scan Spacing of 0.07 mm

**Figure 4:**
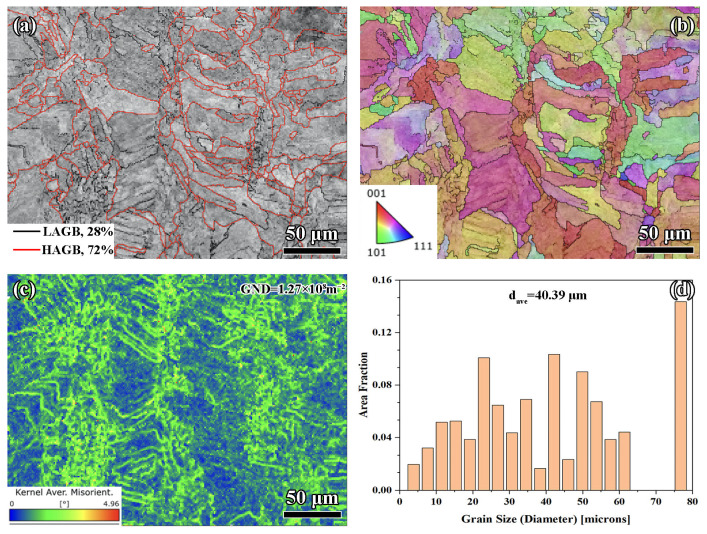
EBSD analysis for 0.07 mm: (**a**) grain boundaries, (**b**) IPF, (**c**) KAM, and (**d**) grain distribution.

As shown in Figure 4a, the EBSD analysis indicated that 72% of the grain boundaries were high-angle grain boundaries (HAGBs), with the remaining 28% being low-angle grain boundaries (LAGBs). The dominance of HAGBs reflected effective recrystallization and grain boundary migration during the PBF-LB/M process [32,33,34], driven by the high thermal gradients and rapid solidification associated with a 0.07 mm scan spacing. HAGBs were energetically favorable sites for dislocation annihilation and were typically associated with improved ductility and crack resistance [35,36,37], as they hindered the transmission of dislocations. However, the presence of 28% LAGBs suggested localized thermal stress accumulation during processing [38]. These LAGBs arose from subgrain formation due to incomplete recovery and could act as sites of dislocation pile-up, contributing to strain localization under cyclic or high-stress conditions [39,40,41].

The inverse pole figure (IPF) is shown in Figure 4b, revealing an isotropic grain orientation distribution, with no strong crystallographic texture observed. This weak texture suggested that the solidification process had been governed by relatively uniform thermal gradients and melt pool dynamics across the layer, despite the high overlap inherent to the 0.07 mm spacing. The isotropy in grain orientation was advantageous for applications requiring uniform mechanical properties in all loading directions, yet the lack of a preferential texture may have reduced yield strength along specific loading directions compared to materials with a controlled fiber or columnar texture.

The kernel average misorientation (KAM) map highlighted significant local misorientations, particularly at grain boundaries and subgrain regions, indicating the presence of high geometrically necessary dislocation (GND) density, measured at 1.27 × 10^8^ m^−2^ (see Figure 4c) using Equation (1) [42]. This elevated GND density was a direct consequence of the rapid cooling and high thermal gradients during the PBF-LB/M process, which promote the generation of dislocation networks to accommodate localized plastic strain [43]. While high dislocation density enhances strength through dislocation hardening, it also introduces strain gradients that could act as potential crack initiation sites under cyclic loading conditions.

(1) $$\rho^{GND}=\frac{2\theta }{\mu_{p}b}$$

where *θ* represents the misorientation angle, *μ_p_* is the pixel, and *b* is regarded as the Burgers vector.

The grain size distribution exhibited a relatively broad range, with an average grain size (*d_ave_*) of 40.39 µm (see Figure 4d, containing 44 grains). The smaller grain size observed in the 0.07 mm spacing sample was consistent with the rapid solidification rates resulting from high energy density and substantial track overlap. Fine grains could improve mechanical strength via the Hall–Petch relationship [44] (see Equation (2)), where smaller grains provided more boundaries to impede dislocation motion. However, the broad grain size distribution observed in this analysis could have led to heterogeneity in mechanical properties, particularly under tensile or compressive loading. The coexistence of finer grains with larger grains may have created localized stress concentration zones, reducing uniformity in deformation behavior.

(2) $$\sigma_{ys}=\sigma_{dr}+\left(k_{y}/\sqrt{d_{ave}}\right)$$

where *σ_dr_* represents the total (frictional) resistance of dislocations and *k_y_* represents the pinning constant.

#### 3.2.2. Scan Spacing of 0.11 mm

**Figure 5:**
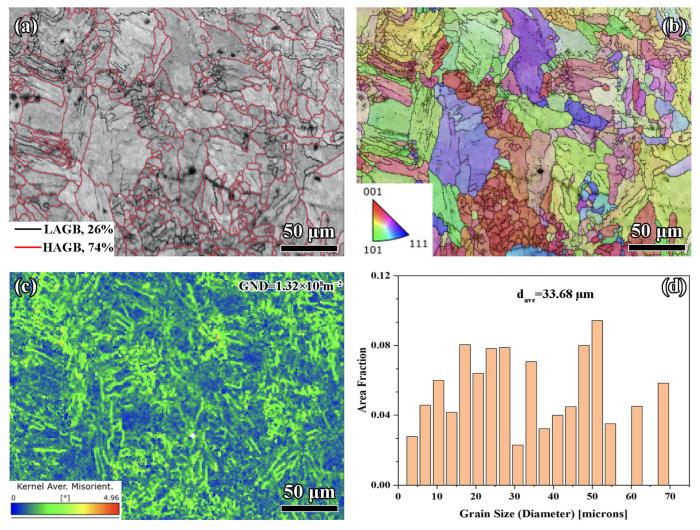
EBSD analysis for 0.11 mm: (**a**) grain boundaries, (**b**) IPF, (**c**) KAM, and (**d**) grain distribution.

The 0.11 mm scan spacing exhibited a higher proportion of HAGBs, accounting for 74% (see Figure 5a), compared to the 72% observed at 0.07 mm. The increased HAGB fraction suggested more effective recrystallization and grain refinement due to improved energy distribution and reduced thermal stress. This higher HAGB content enhances dislocation mobility, contributing to better ductility and resistance to fracture initiation.

Figure 5b shows a similarly isotropic grain orientation as the 0.07 mm spacing but with slightly larger and more uniformly distributed grains. This improved uniformity reflected a more stable solidification process, attributed to the optimized energy input at 0.11 mm spacing. The balanced thermal conditions minimized thermal gradients, reducing the likelihood of residual stresses and microstructural anisotropy.

Figure 5c indicates lower localized strain compared to 0.07 mm, with a GND density of 1.32 × 10^8^ m^−2^, slightly higher than that of the 0.07 mm spacing. This increase in dislocation density reflected enhanced strain hardening capacity without introducing significant strain gradients. The uniform distribution of dislocations supported better mechanical performance under tensile and cyclic loading conditions [45].

Figure 5d presents the grain size distribution at 0.11 mm (containing 72 grains), showing a more refined structure with *d_ave_* of 33.68 µm, smaller than the 40.39 µm observed at 0.07 mm. The finer and more evenly distributed grains enhance strength through the Hall–Petch effect (see Equation (2)) while maintaining ductility. The reduced average grain size also indicates that the optimized energy density of 0.11 mm spacing achieved more consistent thermal management during processing.

#### 3.2.3. Scan Spacing of 0.15 mm

**Figure 6:**
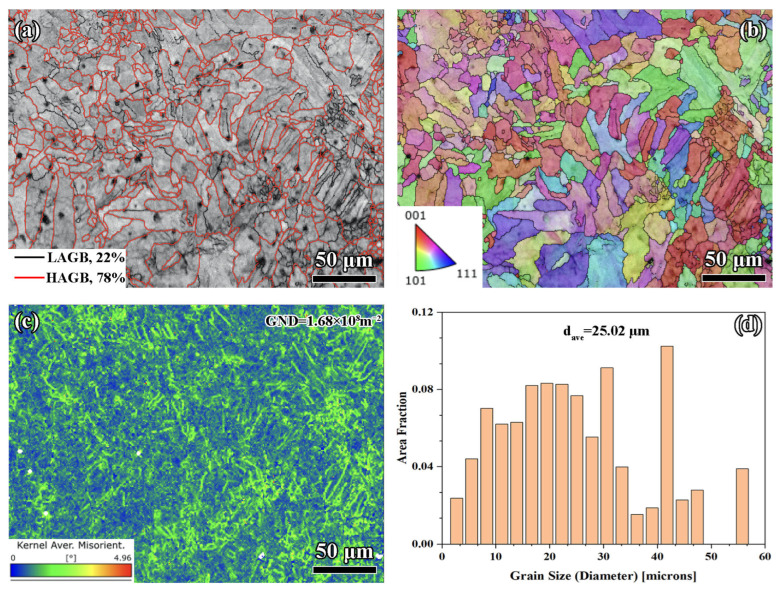
EBSD analysis for 0.15 mm: (**a**) grain boundaries, (**b**) IPF, (**c**) KAM, and (**d**) grain distribution.

The 78% HAGBs observed in Figure 6a indicate a more extensive recrystallization process compared to smaller scan spacings. The reduced energy density at 0.15 mm spacing results in less localized overheating, which promotes stable grain boundary migration and the formation of refined, energetically favorable HAGBs. As shown in Figure 6b, the preferential grain alignment reflects a stronger crystallographic texture, a result of directional solidification driven by stable and consistent thermal gradients. At 0.15 mm spacing, the reduced overlap minimizes thermal fluctuations between adjacent tracks, leading to more uniform cooling rates. As shown in Figure 6c, the GND density reached 1.68 × 10^8^ m^−2^, which is the highest among all tested spacings.

The larger track spacing introduces localized thermal stresses during solidification, which generate higher dislocation densities. Figure 6d indicates that the *d_ave_* was 25.02 µm (containing 102 grains), which was ascribed to the lower energy density and more rapid solidification associated with 0.15 mm spacing. The reduced overlap ensures a steeper thermal gradient, which accelerates nucleation and restricts grain growth during solidification.

Despite the apparent advantages of the 0.15 mm scan spacing as indicated by EBSD, the SEM observations revealed critical defects that undermine its potential mechanical benefits. These defects include significant porosity, a lack of fusion, and unmelted powder particles, resulting from insufficient track overlap. In addition, the dislocations were pinned at defect interfaces to form local pile-ups, which not only failed to achieve effective strengthening through long-range slip but also exacerbated crack initiation due to the amplification of stress concentration. As the main failure source, the stress concentration at the tip of pores generated a fracture driving force far exceeding the plastic deformation capacity of dislocation slip, causing the material to undergo unstable fracture before yielding. Therefore, the “hazard priority” of defects significantly overshadowed the gain from dislocation strengthening [46]. Process optimization was required to reduce porosity and regulate the uniform distribution of GND, so as to achieve the synergistic strengthening of the microstructure.

### 3.3. Tensile Performance

**Figure 7:**
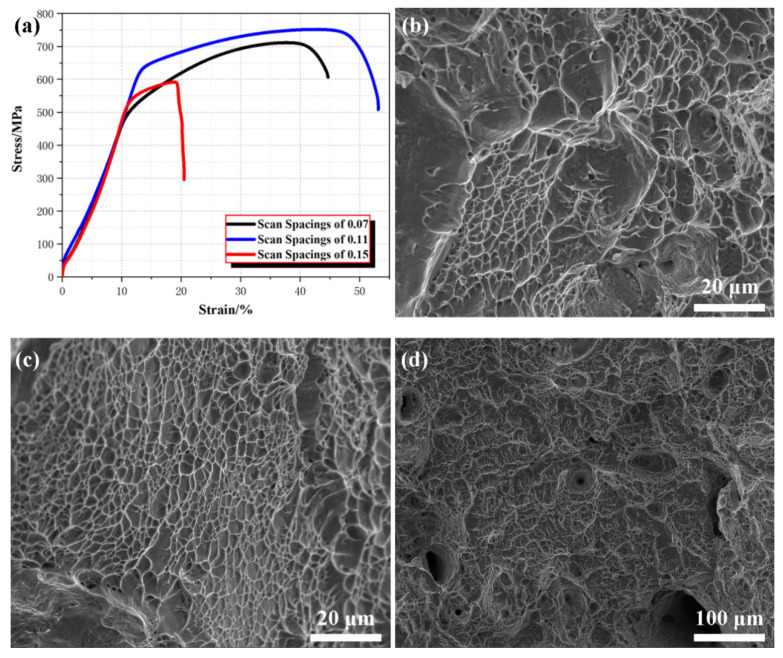
Tensile performance for PBF-LB/M 316SS: (**a**) stress–strain curves under various scan spacings; (**b**) fracture surface of 0.07 mm; (**c**) fracture surface of 0.11 mm; (**d**) fracture surface of 0.15 mm.

The tensile stress–strain curves (Figure 7a) clearly demonstrate the differences in mechanical performance among the three scan spacings. The sample with a scan spacing of 0.11 mm exhibited the highest ultimate tensile strength (UTS, approximately ~750 MPa) and the highest fracture elongation at 53%, reflecting an optimal balance between strength and ductility. This performance is attributed to the uniform microstructure and minimized defects achieved with this spacing.

In contrast, the sample fabricated at 0.07 mm showed a comparable UTS (around 700 MPa) but lower elongation (45%), likely due to residual stresses induced by excessive energy density. The 0.15 mm spacing performed the worst, with a UTS of 590 MPa and an elongation of only 21%, primarily due to processing-induced defects such as porosity and lack of fusion. The fracture surface of the 0.11 mm sample (Figure 7c) displayed deep and uniformly distributed dimples, characteristic of ductile failure and extensive plastic deformation [47,48]. In comparison, the 0.07 mm sample (Figure 7b) showed finer dimples with localized flat regions, indicating limited ductility caused by thermal stresses. The 0.15 mm sample exhibited a mixed-mode fracture (see Figure 7d), dominated by large voids and unmolten regions, which acted as stress concentrators and led to premature failure.

## 4. Conclusions

(1) SEM and EBSD analyses revealed that scan spacing significantly influenced the microstructure of PBF-LB/M 316L stainless steel. A scan spacing of 0.11 mm exhibited the best balance of energy distribution and thermal conditions, resulting in a uniform and dense microstructure with a higher proportion of high-angle grain boundaries (HAGBs), which enhanced ductility and fracture resistance. In contrast, scan spacings of 0.07 mm and 0.15 mm led to microstructural defects and reduced mechanical properties due to excessive or insufficient energy input.
(2) Although the EBSD results for the 0.15 mm scan spacing indicated the smallest average grain size and the highest GND density, the tensile performance was the weakest, primarily due to the presence of significant porosity. This suggested that, compared to microstructural features, good forming quality, i.e., minimizing defects such as porosity and lack of fusion, was fundamental for ensuring the mechanical performance and structural integrity of parts.
(3) The surface morphology produced at 0.11 mm scan spacing was the smoothest and most uniform, with significantly reduced defects such as porosity and lack of fusion. This uniformity in surface and grain structure directly contributed to the highest ultimate tensile strength (750 MPa) and fracture elongation (53%).

### Statement on Novelty

The PBF-LB/M 316L stainless steel with an ultimate tensile strength of 750 MPa and a fracture elongation of 53% was fabricated using a laser power of 200 W, an exposure time of 80 µs, and a scan spacing of 0.11 mm. Although higher scanning spacing is beneficial for better microstructures, such as higher GND density, and finer grains, it can lead to defects such as pores. The tensile results indicated that better microstructures cannot compensate for the weakening of material mechanical properties caused by metallurgical defects. Therefore, in the manufacturing process, more attention should be paid to the forming quality rather than the microstructure.

## Data Availability

The data presented in this study are available on request from the corresponding author due to privacy.
